# Digital dynamic discrimination of primary colorectal cancer using systemic indocyanine green with near-infrared endoscopy

**DOI:** 10.1038/s41598-021-90089-7

**Published:** 2021-05-31

**Authors:** Jeffrey Dalli, Eamon Loughman, Niall Hardy, Anwesha Sarkar, Mohammad Faraz Khan, Haseeb A. Khokhar, Paul Huxel, Donal F. O’Shea, Ronan A. Cahill

**Affiliations:** 1 UCD Centre for Precision Surgery, University College Dublin, 47 Eccles Street, Dublin 7, Ireland; 2 Department of Medical Physics, Mater Misericordiae University Hospital, Dublin, Ireland; 3 Department of Surgery, Mater Misericordiae University Hospital, Dublin, Ireland; 4 Department of Chemistry, Royal College of Surgeons in Ireland, Dublin, Ireland; 5 MathWorks®, Galway, Ireland

**Keywords:** Colon cancer, Rectal cancer, Computational science, Optics and photonics

## Abstract

As indocyanine green (ICG) with near-infrared (NIR) endoscopy enhances real-time intraoperative tissue microperfusion appreciation, it may also dynamically reveal neoplasia distinctively from normal tissue especially with video software fluorescence analysis. Colorectal tumours of patients were imaged mucosally following ICG administration (0.25 mg/kg i.v.) using an endo-laparoscopic NIR system (PINPOINT Endoscopic Fluorescence System, Stryker) including immediate, continuous in situ visualization of rectal lesions transanally for up to 20 min. Spot and dynamic temporal fluorescence intensities (FI) were quantified using ImageJ (including videos at one frame/second, fps) and by a bespoke MATLAB® application that provided digitalized video tracking and signal logging at 30fps (*Fluorescence Tracker App* downloadable via MATLAB® file exchange). Statistical analysis of FI-time plots compared tumours (benign and malignant) against control during FI curve rise, peak and decline from apex. Early kinetic FI signal measurement delineated discriminative temporal signatures from tumours (n = 20, 9 cancers) offering rich data for analysis versus delayed spot measurement (n = 10 cancers). Malignant lesion dynamic curves peaked significantly later with a shallower gradient than normal tissue while benign lesions showed significantly greater and faster intensity drop from apex versus cancer. Automated tracker quantification efficiently expanded manual results and provided algorithmic KNN clustering. Photobleaching appeared clinically irrelevant. Analysis of a continuous stream of intraoperatively acquired early ICG fluorescence data can act as an in situ tumour-identifier with greater detail than later snapshot observation alone. Software quantification of such kinetic signatures may distinguish invasive from non-invasive neoplasia with potential for real-time in silico diagnosis.

## Introduction

Direct visualization by endoscopy is an early component of clinical pathways for many patients with visceral cancer that directs subsequent care. At operation, direct inspection assists quantification of overall cancer burden especially in areas poorly seen by non-invasive workup and impacts surgical strategy. However, determination of tissue nature by operator judgement alone or indeed by traditional biopsy (which takes time, risks sampling errors and distorts residual tissue) is imperfect^1^. Improved intraprocedural tissue characterisation could allow better in situ primary tumour profiling and confirmation/exclusion of metastatic deposits acuminating cancer outcomes and healthcare efficiency^2^.

Surgical colorectal cancer care is ideally suited for digital advance with both endoscopy and laparoscopy embedded in its diagnosis and therapy. Its endolaparoscopic systems are capable of providing multispectral illumination including tissue-penetrating near-infrared (NIR) spectral vision^3^. Such capacity can help characterise tissue intraoperatively with most clinical application thus far for tissue perfusion assessment using the non-selective fluorophore indocyanine green (ICG)^4–6^. For this, the NIR-ICG visualization is dynamic, occurring within seconds of agent administration, fitting current surgical practice very well. In contrast, NIR cancer identification strategies have concentrated on advanced preprocedural fluorophore administration with visual interrogation planned for many hours or days later at a time when tissue agent concentrations are relatively static^7^.

Malignant tumours distinguish from normal tissue and benign neoplasia by hallmark metabolic behaviours including their neovascularization (angiogenesis) as well as cell–cell and host-tumour interstitial interactions^8,9^. Their irregular vascular architecture and lack of a lymphatic recovery system leads to the tumoritropic accumulation of macromolecules such as albumin in solid tumours (in contrast to their rapid clearance in normal tissue)^10^. Such characteristics mean even a chemically non-selective perfusate, such as ICG, could functionally discriminate cancer at any site allowing in situ optical delineation. However, when applied simply by preoperative administration and later snapshot observation, high false positive rates pertain (a problem that also affects cell-targeting agents deployed similarly)^11^.

We hypothesized that kinetic characterisation of the evolving dynamic fluorescence intensity (FI) signature at and within cancer deposits could provide discrimination of cancer versus non-cancer even with a passive, non-targeted fluorophore such as ICG^12^. As the processes being visualized are more complex than perfusion alone and non-tumour background noise may manifest, video-based analytics could de-risk image interpretation permitting greater interpretative clarity and even potentially a ‘digital tissue biopsy’ through software. Herein we pilot the use of systemic NIR-ICG as a licensed available-now agent for endoscopic colorectal neoplasia perfusion interrogation with the goal of identifying discriminant tissue characterisation features. The automated operative perfusion quantification methodology developed and used for this study is available for download so others may test and add to this exploratory work while we continue to expand our clinical series on a collaborative basis.

## Methods

This clinical experience involved adult patients already undergoing endolaparoscopic diagnostic or therapeutic procedures for colorectal neoplasia under general anaesthesia. Informed consent was obtained from all participants in accordance with relevant guidelines and, regulations and with institutional (Mater Misericordiae University Hospital, Dublin, Ireland) ethics approval (1/378/2092, ClinicalTrials.gov Identifier: NCT04220242). ICG is licensed internationally for tissue perfusion characterisation and has an excellent safety profile. NIR Endoscopic Systems are commercially available with the one used in this study (PINPOINT Endoscopic Fluorescence System, Stryker Corp, Kalamazoo, MI, USA) being capable simultaneous white light and NIR display. Therefore, no experimental agents or devices were used and no clinical care decisions changed based on NIR-ICG appearances.

### Clinical study

Patients with colorectal tumors were administered ICG intravenously at a dose of 0.25 mg/kg intraprocedurally. The endoscopic NIR system was used to directly visualise rectal tumours continuously transanally for up to 20 subsequent minutes and enabled a video recording to be made of endoscopic white light and NIR appearances simultaneously. Ex vivo resection specimens from a separate cohort also receiving the same dose of ICG systemically were opened and their mucosal surfaces examined post-operatively with the same camera for still image analysis. In addition, to examine for photobleaching effects in tissue, two opened specimens were examined continuously postoperatively for 20 min at the site of neoplasia and the recording analysed similarly to the in situ rectal recordings (see below).

### Data collection

The videos along with similar high-resolution photographs of the opened mucosal surface of any resected specimens ex vivo underwent post-hoc fluorescence intensity (FI) analysis. All imagery contained both the neoplastic lesion and some adjacent normal tissue. Patient demographics and reactions possibly related to ICG usage were recorded prospectively along with the routine pre and postoperative pathological and radiological reports to capture cancer-relevant details.

### ICG static image analysis

**Figure 1:**
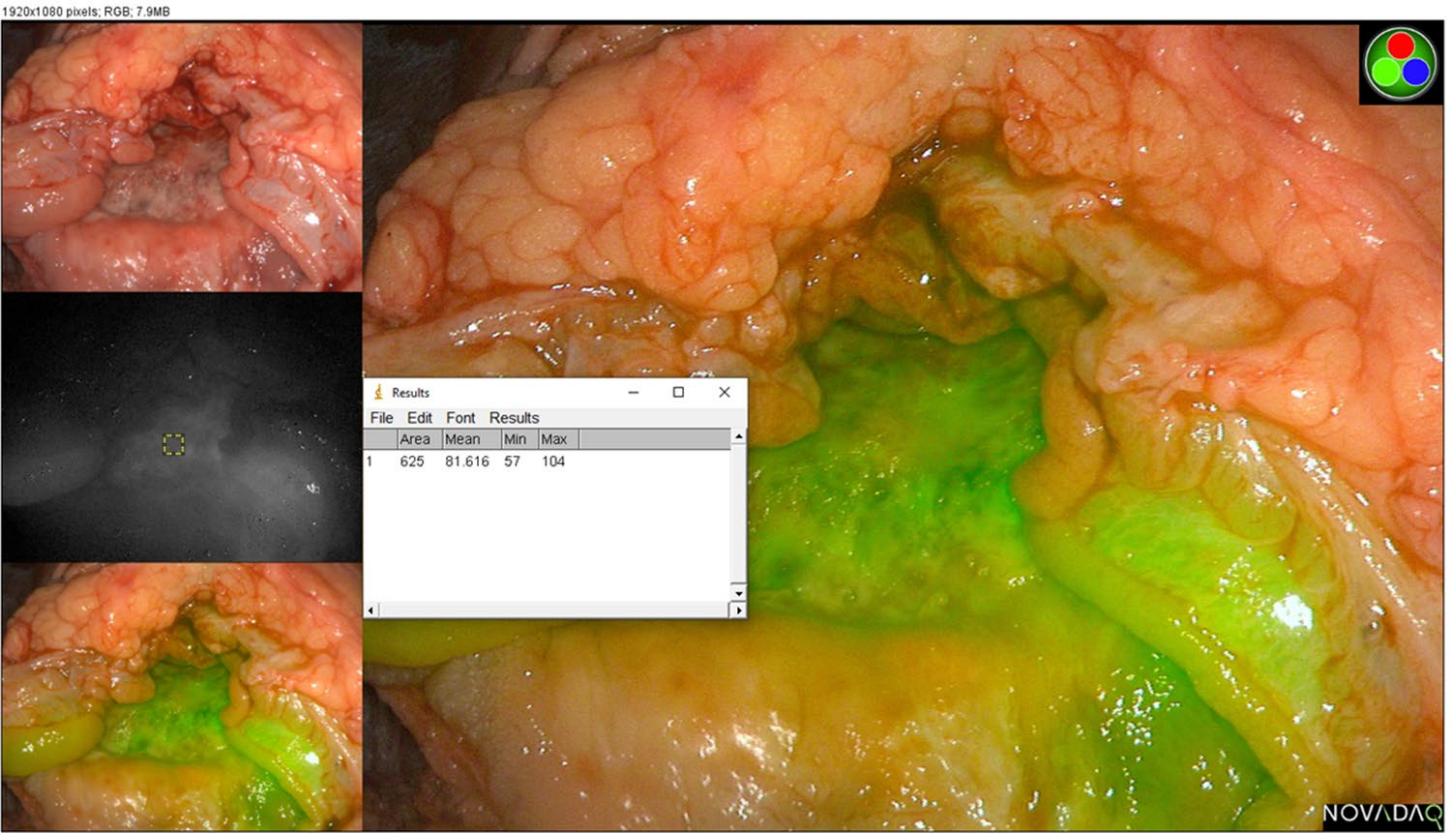
Photograph of mucosal surface of an ex vivo opened, resection specimen in a patient with rectal adenocarcinoma being assessed for tumour static signal intensity using ImageJ. ICG was administered at the time of surgery commencement with the photograph being taken postoperatively using the Pinpoint endoscopic NIR system.

Fluorescence photographs of resected neoplastic specimens were processed post-hoc using ImageJ (NIH, USA)^13^ after annotation of regions of interest (ROI) on the endomucosal surface regions representing the tumour and adjacent normal tissue (control) avoiding obvious areas of surface blood by a surgeon. Spot mean FI in 25 × 25 pixel areas in the tumour were contrasted with normal mucosa (see Fig. 1).

### ICG dynamic data analysis

**Figure 2:**
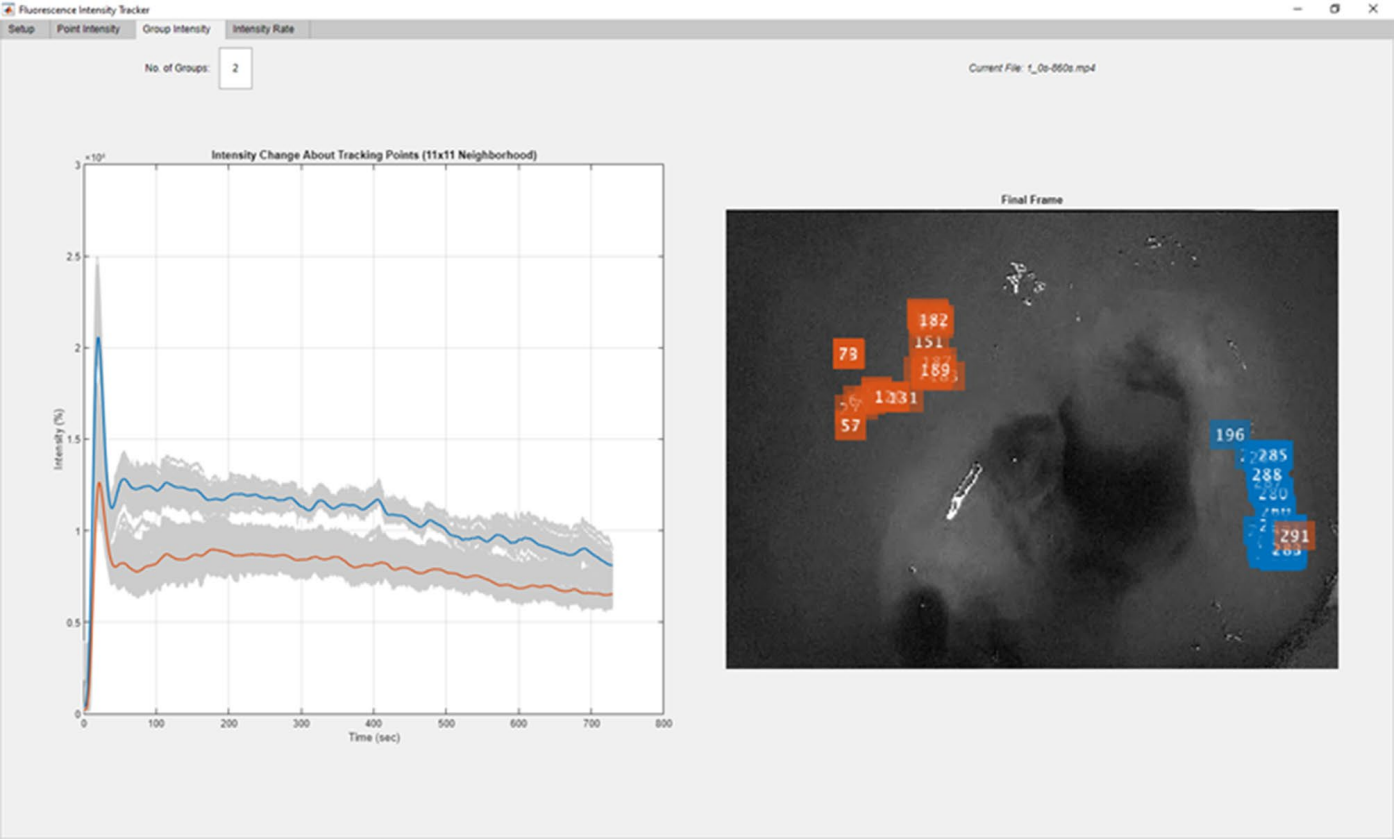
Screenshot of Fluorescence Tracker App (FTA, MATLAB^®^) showing post-hoc automated video-based classification of tumour (blue cluster) versus control tissue (orange cluster) once the regions of interest have been defined via initial annotation by a surgeon on the video frame corresponding to the time of fluorescence inflow. Videos are taken in vivo endorectally using the Pinpoint endoscopic NIR laparoscope while injecting 0.25 mg/kg ICG.

For recorded videos (all rectal tumors and the two postoperative specimens being examined re photobleaching effects), a surgeon again postoperatively annotated ROIs as above also avoiding those areas only variably seen over time in the movies. Post-hoc kinetic plotting of FI dynamic patterns of in situ lesions over time, was initially performed using Image-J on grayscale still image stacks serially sampled at one frame per second (fps) from the time of fluorescence signal onset to video end. Thereafter fluorescence measurement was digitalized and automated through the creation of a bespoke, standalone Fluorescence Tracker App (FTA), developed in collaboration with MathWorks^®^, Galway, Ireland (software source code and executable now publicly available via the MATLAB^®^ File Exchange, www.mathworks.com). This allowed in-app surgeon-ROI-annotation for tumour and control areas from the white-light (RGB) images prior to the onset of non-zero fluorescence intensities in the NIR images with automatic detection and frame-by-frame tracking of surface feature points in the white light images (using minimum eigenvalue^14^ and Kanade–Lucas–Tomasi algorithms respectively^15^) to account for the changing pixel locations of these areas due to camera movement. By continuously tracking in the white-light image, the app maps FI in the corresponding pixels of the complimentary NIR image for the ROIs throughout the video duration. Also, following surgeon annotation of tumour versus control ROI, the software can group the dynamic intensity results using K-Nearest Neighbour (KNN) algorithm (see Fig. 2). For automated analysis, five minutes of continuous signal quantification was set as the minimum goal. In the event that tracking was compromised (e.g. due to instruments obstructing the tracking points in the field of view or large camera movements forcing the points out of frame), ROIs were re-selected, automatically featured, and tracking resumed. Interruptions in individual patient readings were collated and gaps documented.

### Statistical methods

**Figure 3:**
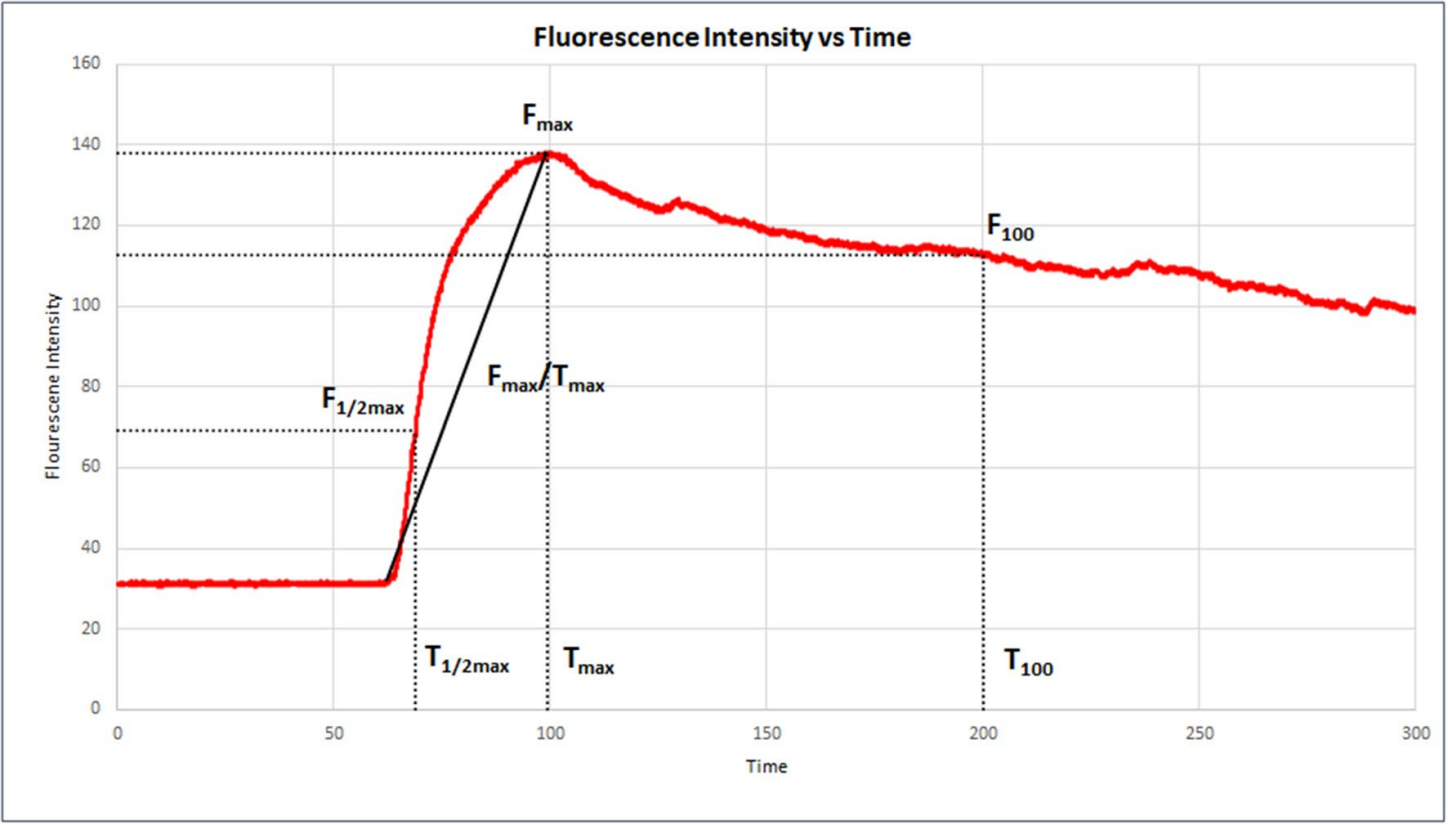
Representative graph (Microsoft Excel for Microsoft 365) of in vivo NIR intensity patterns plotting fluorescence intensity versus time to show the curve variables selected for statistical comparison between malignant, benign and normal tissue in videos from patients in this series.

Data collection and statistical analysis was carried out using Microsoft Excel for Microsoft 365(Microsoft, Redmond, WA, USA) and SPSS Software (IBM, Armonk, NY, USA) version 26 with statistical significance ascribed to p-values less than 0.05. Spot ex vivo FI measurements and dynamic mean values were compared from tumours versus control tissue using Mann–Whitney U. FI signals from in vivo videos were plotted against time and examined for distinctions regarding specific curve milestones and variables adapted from previously described methodologies^16,17^. An intensity rise of 5 grey units was marked as the end of the latency period and the starting block for the incline. Following the rapid inflow period, the point of maximum fluorescence intensity (F_max_), time to achieve this summit (T_max_) and respective gradient (F_max_/T_max_) were calculated along with the time taken (T_½max_) to achieve half the maximum (F_½max_) intensity. Following peak intensity, fluorescence clearance from the target tissue was flagged at 100 s (T_100_) from the start of the descent (see Fig. 3). Final intensity, gradients, duration of tracking, skew and kurtosis were transcribed or calculated. Mean intensities for the tumours and controls before and after KNN grouping were compared using the Wilcoxon signed rank test.

## Results

**Figure 4:**
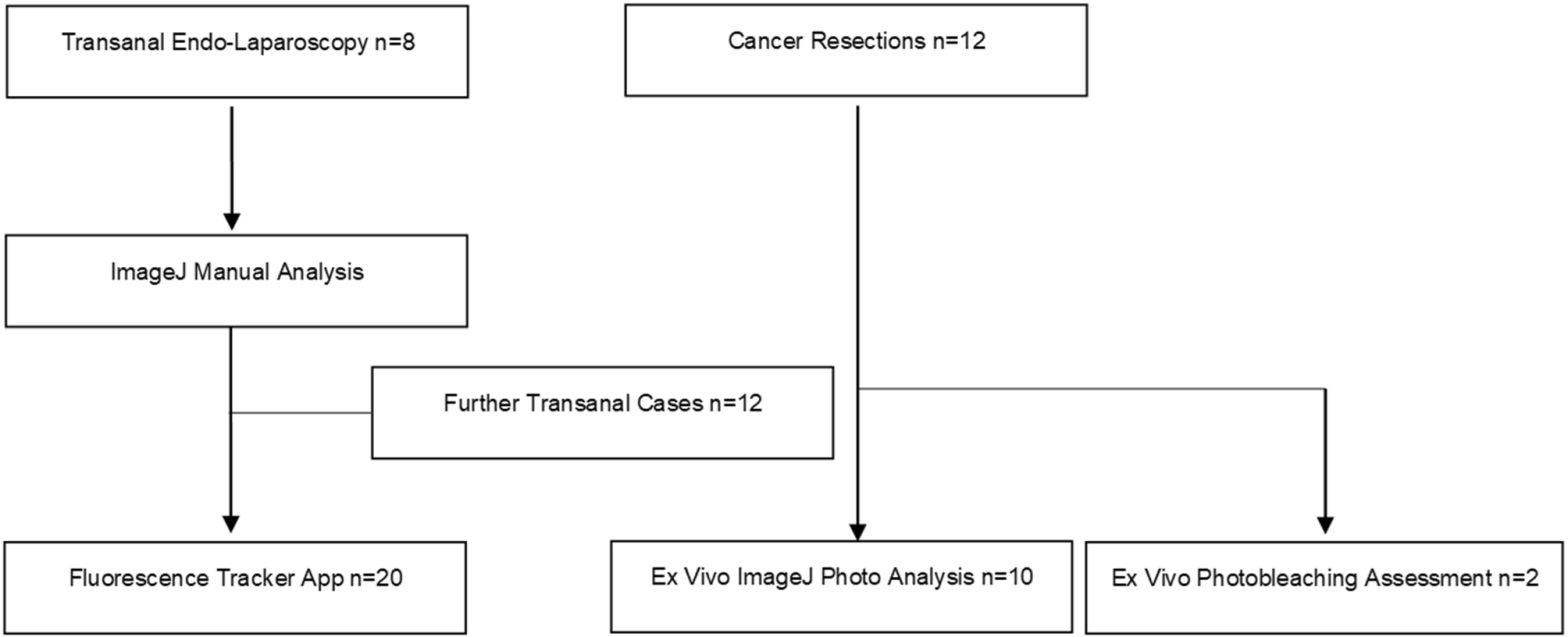
Diagram (Microsoft Word for Microsoft 365) showing cohorts of patients in this study by their methods of fluorescence intensity analysis.

In total, thirty-two patients were recruited into the study (see Table 1 and Fig. 4). No patient suffered ICG reaction or toxicity. All procedures progressed as planned, including diagnostic biopsies and therapeutic endoscopic, laparoscopic and open resections, highlighting that the NIR-ICG assessment was coincidental to patient care. Twelve patients with colorectal cancer had their resected specimens examined postoperatively (therefore 4–6 h post ICG administration). Twenty patients with rectal neoplasia underwent endoscopic primary tumour site assessment under simultaneous white-light and NIR examination immediately after ICG administration continually for up to 20 min.

**Table 1:** Patient and case demographics overall and by analytic methodology.

	In vivo Dynamic video readings (MATLAB®)	Ex vivo spot readings (ImageJ)
n	20	10
Male:female	16:4	7:3
Mean age in years	68.6	62.6
Operation or site	Diagnostic (EUA) = 9 Therapeutic (TAMIS/TART) = 11	Caecum/ascending = 4 Sigmoid/rectum = 6
Benign:malignant	11:9	0:10
Tumour stage by TNM classification	Tx = 1 T1 = 1 T2 = 0 T3 = 6 T4 = 1	Tx = 0 T1 = 1 T2 = 1 T3 = 6 T4 = 2
	Nx = 1 N0 = 4 N1 = 2 N2 = 2	Nx = 0 N0 = 7 N1 = 2 N2 = 1
	Mx = 0 M0 = 7 M1 = 2	Mx = 0 M0 = 8 M1 = 2

Dynamic intensity readings were made by the automated Fluorescence Tracker App. EUA, TAMIS and TART respectively denote Examination under Anaesthesia, Transanal Minimally Invasive Surgery and Transanal Resection of Tumour. In addition, two additional patients with colorectal cancer had their specimens assessed specifically to elucidate the photobleaching impact of continuous illumination over the time-period of the dynamic in-situ recordings (see Figure six).

**Figure 5:**
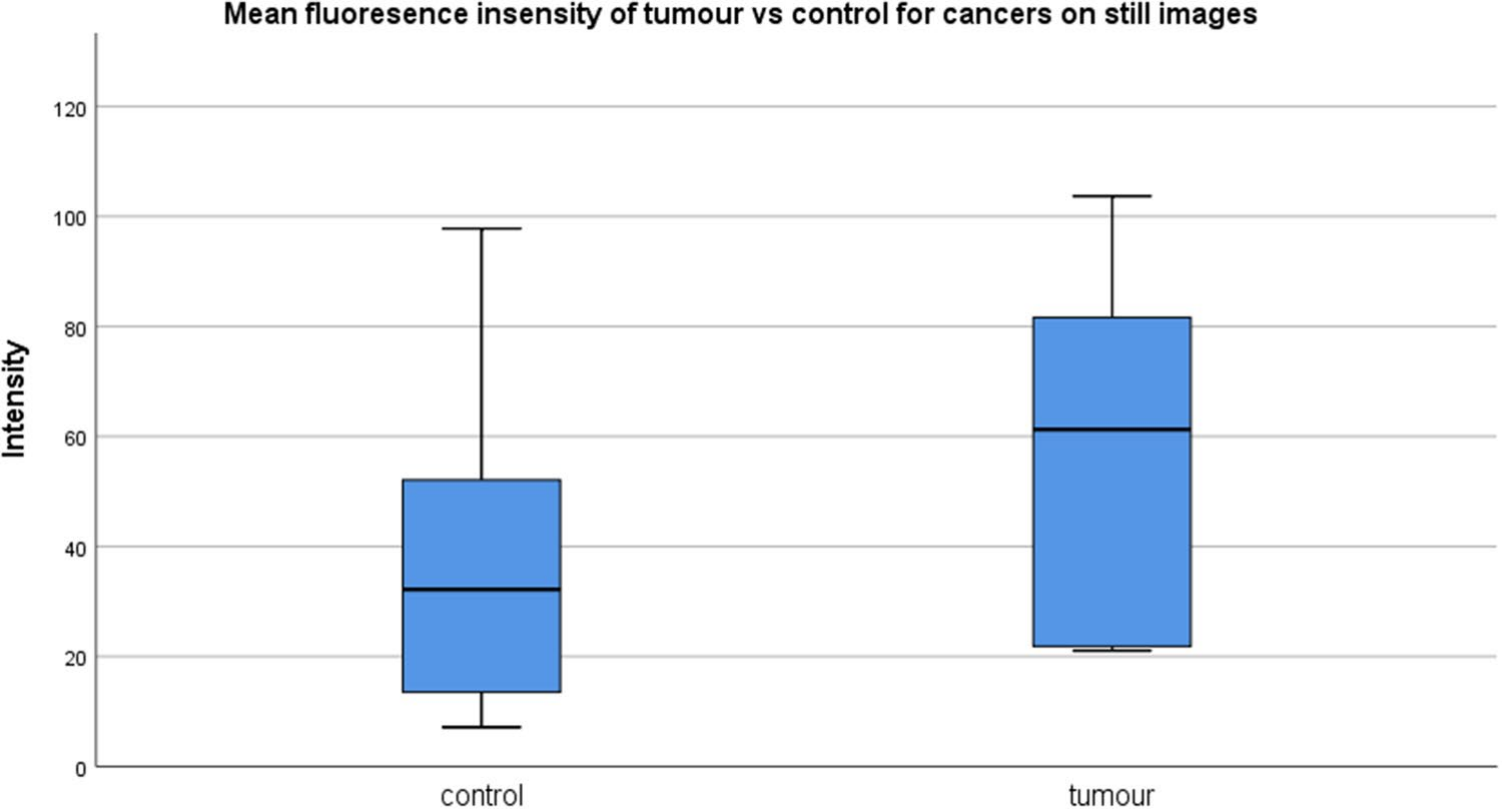
Box plots (SPSS Software version 26) of mean fluorescence mucosal intensity measurements for cancers vs control tissue on postoperative, ex vivo, opened resection specimens via still image analysis with ImageJ in patients receiving ICG at operation commencement.

**Figure 6:**
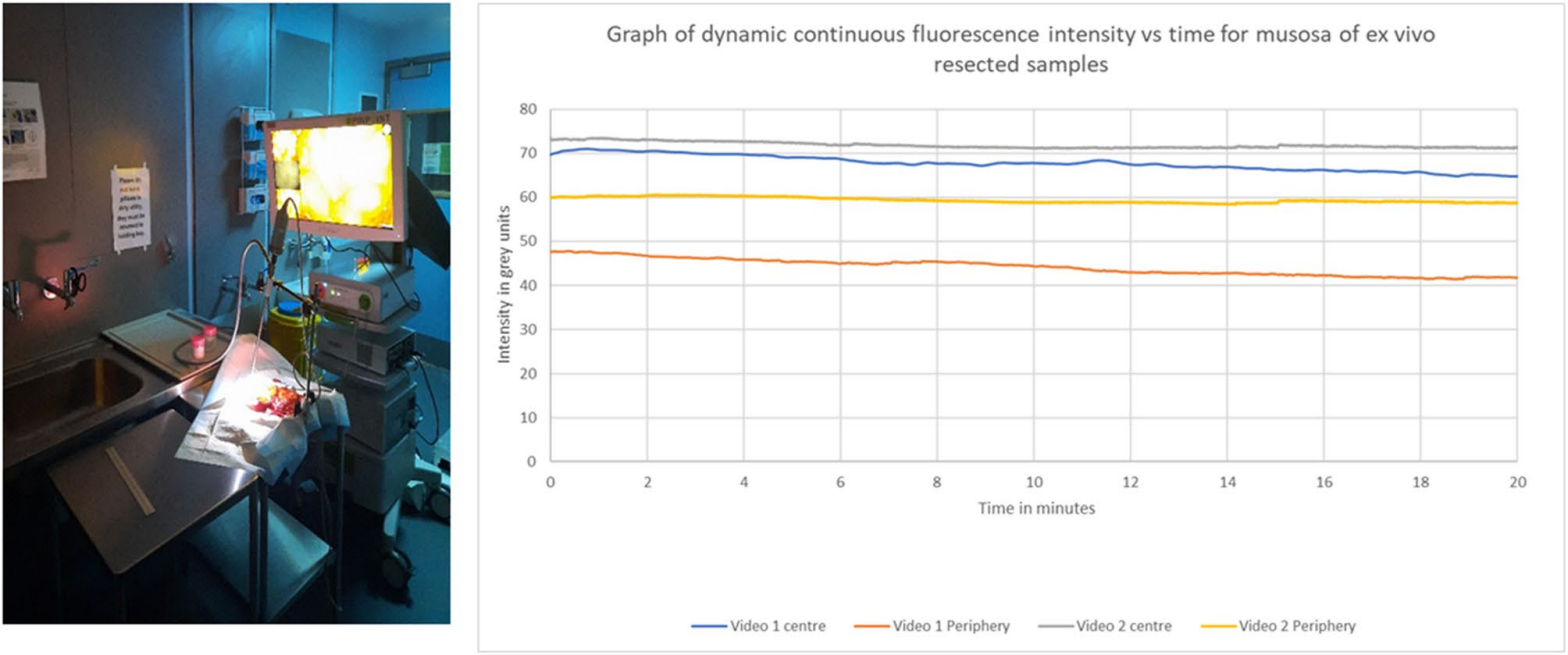
Photograph and graph (Microsoft Excel for Microsoft 365) showing the set-up and impact of continuous illumination on ex-vivo tissue containing cancer hours after systemic administration of ICG in vivo. Postoperatively, the specimen was opened and illuminated with a clamped NIR camera and the video recording tracked continuously to elucidate FI over time with static position and in situ dye concentration. This showed minimal induction of photobleaching both at the centre and peripheries of the image field of view.

With respect to spot ex vivo intensity measurement (n = 10) late after ICG administration, mean (± standard deviation) cancer intensity was non-significantly raised (57.0 ± 61.3) compared with control (36.0 ± 28.3) (Mann–Whitney U) with considerable overlap of scores suggesting poor discrimination of cancer by single point in time analysis even some hours after ICG administration (see Fig. 5). Interestingly, photobleaching was seen to occur to only a minor extent (mean 5%) when examined (n = 2) for in excised specimens using continuous examination of ROIs over twenty minutes (the same period of time tumors were visualized in situ) (see Fig. 6).

**Figure 7:**
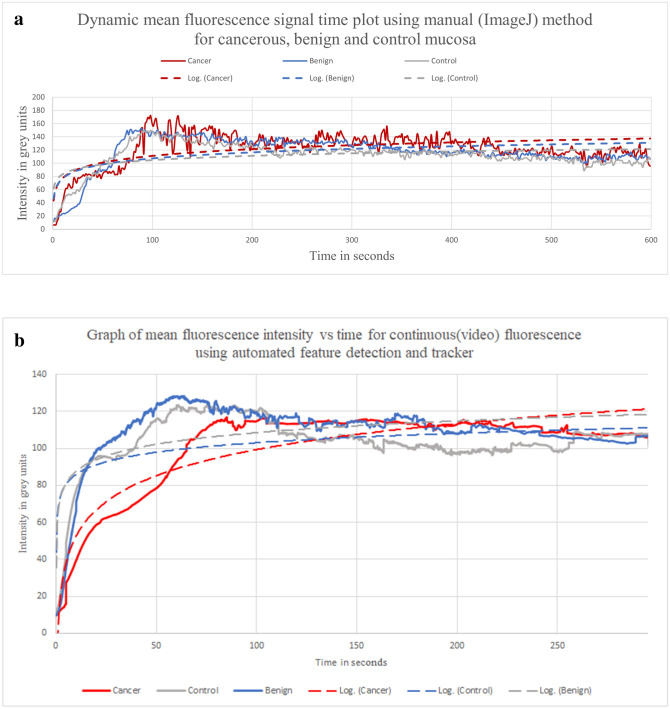
Graph (Microsoft Excel for Microsoft 365) of mean fluorescence intensity per frame vs time for continuous (video) in vivo fluorescence signals using (**a**) manual Image J methodology (1 fps) and (**b**) the Fluorescence Tracker App (30 fps) with automated feature detection and tracker in the video recordings of patients with both benign and malignant rectal lesions. Mean frame intensity for cancer/benign tumours and normal tissue (solid lines) and logarithmic fitted curves (dotted lines).

**Figure 8:**
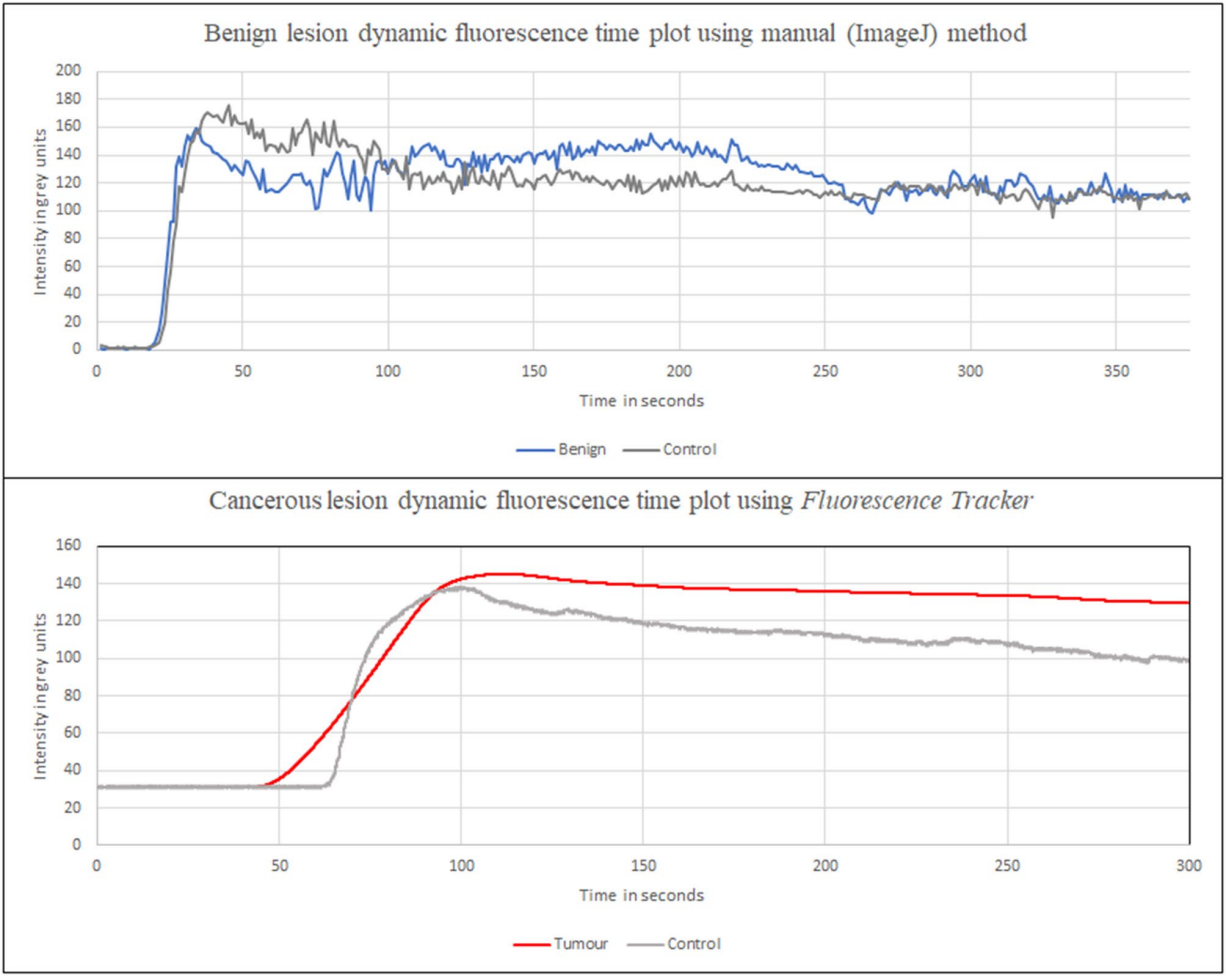
Illustrative example graphs (Microsoft Excel for Microsoft 365) of dynamic time vs fluorescence signal intensity plots for both (**a**) a benign lesion using the manual (ImageJ) method above and (**b**) a cancer using the automated Fluorescence Tracker App for endoluminal in vivo assessment while injecting 0.25 mg/kg ICG.

Regarding dynamic recordings of in-situ lesions, grossly different patterns of ICG flow through cancer versus benign tumors were visually apparent-with dye being slow in ingress into cancers early and seeming to persist for longer once penetrated versus adjacent normal tissue. Re FI, eight recordings (three cancers), were analysed manually by Image J (1 fps) and both these and the next twelve were analysed using the automated FTA. Both manual Image J analysis (n = 8) and automated FTA analysis (n = 20) of lesions in situ being dynamically profiled displayed potentially discriminative statistical differences between cancers and benign tumours from their FI temporal signature kinematics. Figure 7 shows graphs of mean FI over time for such lesions grouped by their nature using both the manual Image J and FTA methodology while Fig. 8 shows for illustration the FI time-plot for a representative benign and malignant lesion. Alongside providing cleaner, richer data due to its higher sampling frequency (30 fps), the FTA method was faster, required less user input and sampled more intensely allowing discriminative curves with shorter video analysis (average continuous FTA tracking time 601 s vs 1903s with manual ImageJ). Furthermore, only 142 of 24,051 s (0.006%) of videos had to be censored due to inability to track serially using the FTA.

Tables 2 and 3 show the measures of graph parameters from the lesions viewed endoscopically by both manual ImageJ and automated FTA FI curves. Statistical analysis of temporal kinetic patterns generated by the manual quantificative method with respect to flow milestones of lesions versus global control readings revealed significant peak intensity differences (p = 0.047, Kruskal Wallis). Direct F_max_ comparisons showed that the cancer intensity curve zenith (F_max_ 202 ± 35.68) was significantly higher than for both the benign tumours (165.8 ± 11.69, p = 0.02) and control tissue (169.63 ± 12.84, p = 0.04) (Mann–Whitney U). With respect to the FTA graphs, three-way analysis using Kruskal–Wallis comparison of cancer, benign and control regions showed significant differences in upslope (gradient from baseline to F_max_ p = 0.039) and for both the downslope and fall between F_max_ and T_100_ (p = 0.046 for each). Direct comparison with Mann–Whitney U between cancers and control regions showed that cancers peaked significantly later, took significantly longer to rise from baseline to maximum and exhibited a shallower gradient to the peak. This resulted in a significantly later T_100_. Skew for cancers was further to the right (− 1.69 ± 1.17 vs − 0.41 ± 1.37 p = 0.04). In comparing cancers with benign lesions, there were also significant differences by T_100_ in terms of both fall and downward gradient. There was no statistically significant difference in these measures between all lesions together and benign lesions alone compared with the controls.

**Table 2:** Tabulated measures of graph parameters from manual ImageJ quantification of fluorescence intensity curves for videos of rectal lesions viewed in vivo, endoscopically under both white light and near-infrared continuously after 0.25 mg/kg ICG administration. g.u denotes grey units.

Variables	Mean values ± standard deviation			Kruskal–Wallis	Mann–Whitney U *p* values		
	Benign (n = 5)	Cancer (n = 3)	Control (n = 8)	p values	Cancer vs control	Benign vs cancer	Benign vs control
**Upslope**							
Intensity at the end of latency in g.u.	16.4 ± 22.14	11.33 ± 10.97	17.25 ± 18.39	0.329	0.22	0.29	0.37
Time at end of latency in s	15.87 ± 11.59	12.1 ± 5.58	21.23 ± 18.93	0.870	0.84	0.88	0.56
F_max_ in g.u.	165.8 ± 11.69	202 ± 35.68	169.63 ± 12.84	0.047*	0.04*	0.02*	0.46
Intensity rise from end of latency in g.u.	149.4 ± 23.46	190.67 ± 41.43	152.38 ± 29.31	0.226	0.15	0.10	0.71
T_max_ in s	60.95 ± 23.56	351.82 ± 307.08	84 ± 43.88	0.116	0.10	0.05	0.46
Time to rise from end of latency in g.u.	45.08 ± 30.56	339.73 ± 312.21	62.77 ± 53.8	0.112	0.07	0.05	0.88
Gradient	5.5 ± 4.34	1.51 ± 1.89	4.7 ± 4.43	0.193	0.10	0.18	0.38
**T**_**50**_							
F_1/2max_ in g.u.	74.7 ± 11.73	95.33 ± 20.71	76.19 ± 14.65	0.226	0.15	0.10	0.71
T_1/2max_ in s	12.77 ± 12.77	69.92 ± 67.88	14.01 ± 11.7	0.166	0.10	0.10	0.56
T_1/2max_/T_max_	0.2 ± 0.18	0.31 ± 0.32	0.2 ± 0.21	0.895	0.68	0.65	1.00
**T**_**100**_							
T_100_ in s	160.95 ± 23.56	451.82 ± 307.08	184 ± 43.88	0.116	0.10	0.05	0.46
F_100_ in g.u.	133 ± 11.85	143.33 ± 39.8	121.13 ± 28.39	0.590	0.41	0.46	0.51
Fall_100_ (intensity decline from peak to F_100_) in g.u.	32.8 ± 7.73	58.67 ± 74.23	48.5 ± 20.17	0.431	0.41	0.46	0.24
DownSlope_100_ gradient	0.33 ± 0.08	0.59 ± 0.74	0.49 ± 0.2	0.431	0.41	0.46	0.24
**Downslope**							
Length of tracking in s	1767.6 ± 154.21	2134.67 ± 153.44	1902.63 ± 240.72	0.084	0.12	0.03*	0.38
Time following T_max_ in s	1706.65 ± 166.86	1782.84 ± 460.45	1818.62 ± 235.59	0.696	0.84	0.65	0.38
Final Intensity in g.u.	46 ± 29.56	64.33 ± 16.86	38 ± 26.94	0.328	0.15	0.37	0.51
Downslope gradient	0.07 ± 0.03	0.08 ± 0.02	0.07 ± 0.02	0.788	0.54	0.65	0.66
**Distribution**							
Kurtosis	0.73 ± 3.32	0.62 ± 1.57	− 0.17 ± 1.23	0.663	0.41	0.46	0.77
Skew	− 0.29 ± 0.87	0.17 ± 0.18	0.19 ± 0.46	0.417	0.84	0.46	0.19

**Table 3:** Tabulated measures of graph parameters using the automated Fluorescence Tracker App (MATLAB®) of fluorescence intensity curves for videos of rectal lesions viewed in vivo, endoscopically under both white light and near-infrared continuously after 0.25 mg/kg ICG administration. g.u denotes grey units.

Variables	Mean values ± standard deviation			Kruskal–Wallis	Mann–Whitney U *p* values		
	Benign (n = 11)	Cancer (n = 9)	Control (n = 20)	p values	Cancer vs control	Benign vs cancer	Benign vs control
**Upslope**							
Intensity at the end of latency in g.u.	14.62 ± 15.14	15.91 ± 10.23	19.95 ± 17.24	0.506	0.96	0.38	0.26
Time at end of latency in s	11.54 ± 14.35	26.43 ± 29.59	17.91 ± 19.85	0.400	0.32	0.24	0.46
F_max_ in g.u.	153.22 ± 23.37	136.43 ± 22.61	144.82 ± 29.37	0.378	0.60	0.14	0.36
Intensity rise from end of latency in g.u.	138.59 ± 27.89	120.52 ± 25.93	124.87 ± 31.45	0.320	0.92	0.16	0.20
T_max_ in s	83.56 ± 89.18	190.18 ± 138.81	72.62 ± 65.88	0.069	0.03*	0.07	0.68
Time to rise from end of latency in g.u.	72.02 ± 92.3	163.75 ± 129.01	54.71 ± 60.51	0.055	0.02*	0.10	0.48
Gradient	4.18 ± 3.39	1.73 ± 1.66	4.44 ± 3.48	0.039*	0.01*	0.05	0.87
**T**_**50**_							
F_1/2max_ in g.u.	69.3 ± 13.95	60.26 ± 12.96	62.43 ± 15.72	0.320	0.92	0.16	0.20
T_1/2max_ in s	11.8 ± 6.74	11.24 ± 6.28	13.19 ± 11.8	0.974	0.96	0.97	0.77
T_1/2max_/T_max_	0.21 ± 0.11	0.15 ± 0.15	0.24 ± 0.13	0.172	0.07	0.21	0.48
**T**_**100**_							
T_100_ in s	183.56 ± 89.18	290.18 ± 138.81	172.62 ± 65.88	0.069	0.03*	0.07	0.68
F_100_ in g.u.	109.36 ± 30.77	111.37 ± 19.9	106.98 ± 34.68	0.808	0.51	0.91	0.71
Fall_100_ (intensity decline from peak to F_100_) in g.u.	43.86 ± 18.67	25.07 ± 16.15	37.84 ± 18.52	0.046*	0.09	0.02*	0.23
DownSlope_100_ gradient	0.44 ± 0.19	0.25 ± 0.16	0.38 ± 0.19	0.046*	0.09	0.02*	0.23
**Downslope**							
Length of tracking in s	612.19 ± 354.53	595 ± 320.69	598.09 ± 334.76	0.970	0.98	0.91	0.79
Time following T_max_ in s	528.63 ± 339.73	404.82 ± 372.37	525.47 ± 368.56	0.396	0.24	0.21	0.84
Final intensity in g.u.	82.75 ± 48.13	94 ± 31.18	78.15 ± 43.13	0.560	0.26	0.57	0.77
Downslope gradient	0.16 ± 0.1	0.2 ± 0.22	0.21 ± 0.26	0.542	0.30	0.38	0.79
**Distribution**							
Kurtosis	5.12 ± 6.77	6.51 ± 7.92	2.68 ± 4.17	0.352	0.13	0.52	0.59
Skew	− 0.89 ± 1.72	− 1.69 ± 1.17	− 0.41 ± 1.37	0.107	0.04*	0.24	0.32

Within the group analysed by FTA, six cancer cases underwent application of the KNN classification algorithm. With surgeon ROI (lesion and control) selection, the algorithm was able to cluster the data into two groups segregating cancer from normal tissue automatically, in a fashion not significantly different to the manually segregated data (Wilcoxon signed rank resting). However, the automated classification was unable to reproduce the significantly raised ‘*time to rise*’ demonstrated on neoplastic tissue (p = 0.041) versus control when these were manually grouped.

## Discussion

Fluorescence-guided surgery is proposed as the next progressive iteration in cancer surgery, especially endo-laparoscopic operations^2,3^. Notably, it is already impacting decision-making and step-sequencing in reconstructive surgery both of the gastrointestinal tract^18^ and other tissues^19^. As well as a means to demarcate watersheds between perfused and non-perfused intestine, other uses include critical boundary identifications such as ureters^20,21^ and fascial planes^22^ by direct instillation. For cancer and draining lymph node identification, ICG has been placed peritumorally submucosally by endoscopic tattoo^23–25^. In situ revelation of cancer including tumour margination however requires labelling pathology rather than physiology. While cancerous tissue can retain fluorescent agents, there remains a significant issue with false positive findings^26,27^. Because of this lack of sensitivity, most research groups are now focusing on development of new cancer-selective agents although accuracy remains problematic^11,28–30^. Given that cancers likely act to accumulate agents in their vicinity differently to other areas that trap agents more passively, we thought to observe dynamically the processing of ICG by tumours under continuous direct observation different to others who have focused on single point in time observations.

In this experience, digital FI quantitative assessment of tumour perfusion angiograms has been demonstrated, controlling too for potential impact due to photobleaching (a well described phenomenum in vitro^25,31^ but one that seems less relevant in vivo due perhaps to protective effect of tissue and dynamic turnover of dye in the ROI in situ). This desktop post-hoc based assessment of the curve metadata has demonstrated possible discriminative flow milestones and timepoints related to the early phase behaviour of the dye in the peritumoral environment that preludes late stage appearances. While additional distinguishing parameters may also exist and could be identifiable via longer videos enabling analysis of dye processing and efflux patterns and/or by using more sophisticated analytical methods, this initial work is encouraging (and indeed longer in situ observation periods may be clinically impractical).

Overall, the fluorescence signal in cancers takes longer to peak and retains this intensity longer (consistent with impaired local pharmacokinetics compared with healthy tissue). Such a distinct FI signature could be visually noticed in both grey-scale, composite and threshold modes and could be quantified with post-hoc image data analysis. Manual intensity quantification allowed mathematical comparisons of the FI time patterns although such data generation was labour-intensive while episodic sampling (even at 1fps) made smoothing of data difficult and background noise impaired statistical analysis. Automating feature detection, tracking and quantification enabled higher sampling rates (30fps) and so provided richer data for analysis with processing time being a function of computational ability rather than human labour.

While further work needs to be done these findings are of great potential clinical relevance. The ability to optically characterize neoplasia through its biological hallmark behaviours could help optimize clinical diagnostic pathways by immediately indicating the nature of lesion and facilitate, in cases of benign aetiology, immediate local excision (or conversely caution such an approach in lesions with benign appearances but containing an invasive focus). Avoidance of biopsy is increasingly advocated in these cases as such an act can introduce architectural distortion in the samples and induce fibrosis in the residual lesion (confounding luminal excision)^32^. In lesions where biopsy is still indicated, the fluorescent signal profile could indicate the site of most relevant yield re cancer detection. With regard to visible lesions, the key issue to discriminate is cancer versus not cancer and so benign tumor behaviour being similar to normal tissue fits the clinical paradigm re lesion management.

To be maximally useful however this work of course necessitates future effort translating our dataset into a classifier which would allow validation against a gold standard such as histopathology. In this regard, KNN clustering showed encouraging discriminative abilities and which may be refined with further artificial intelligence methodologies^33^. Detailed region by region biopsy collated to continuous FI perfusion criteria may too yield digital tumour margin delineation and heterogeneity appreciation. Greater patient numbers is of course needed too and, with early proof of principle now provided, a larger clinical study is now ongoing to develop and validate these findings ^32^ while AI methods are also being developed to enable real-time deployment^34^.

Cell-specific targeting remains of great interest for future advances in this area but a deep understanding of tumoural functional characteristics is necessary to best inform the purpose and level of probe engineering needed. ICG accumulates quickly and can be effectively imaged with a variety of commercial cameras and so there is much to be learned from this study for new agent development. Its non-specific tumour uptake mechanisms include enhanced permeability and retention (EPR), aberrant gap junctions and dysregulated pinocytosis^35–37^. As ICG can be seen in this experience to passively concentrate in tumours, it seems likely that so too will, at least in part, a “cancer-specific” agent. Such internalisation mechanisms could prove advantageous in performance over cell-surface or ligand-specific agents which could be limited in applicability due to variance in intra and inter-tumour target expression. It is likely also that the ICG uptake mechanisms through cancer pathophysiology seen here are shared across other common cancers and could extend to sites of regional spread and metastatic disease.

While cancer cell selectivity can be engineered in cell culture, tumour imaging in vivo encompasses a variety of host:cancer interactions as well as additional cell signalling and response pathways. Furthermore, additional unrelated sites of inflammation^38^ or injury will equally be prone to incidental, unintended probe accumulation. This indicates that a relatively high rate of false positive findings may continue despite any additional tumour targeting features. Rigorous preclinical and clinical study controls are required to minimize heuristic mechanistic targeting assumptions. While long-in-advance (24–48 h) administration of the fluorophore has been proposed to offset non-cancer signalling (and allow signal strengthening by agent concentration), a significant downside exists with respect to the unpredictability of operative timing and patient-to-patient pharmacokinetic variances.

An inbuilt synchronous and continuous dynamic quantification algorithm and screen display can be of use to all fluorescence imaging agents and could even enable synchronous use of multiple probes of different wavelength colours. Addition of cancer-specific targeting could still accelerate agent accumulation at the ROI following systemic distribution, enabling short-window observations with the greatest sensitivity and specificity possible within minutes. This would be of use for the operator more akin to episodic perfusion assessment with the added biochemical, biological and imaging complexity hidden. “Off–on” fluorogenic compounds are especially attractive for this purpose as their signalling only switches on when they arrive at the appropriate microenvironment^39^.

This study is of course limited by the small cohort studied although has the strength that patients were recruited into the study without arbitary preselection. Fluorescence data is also dependent on the recording of the detected fluorescence signal. Video stability, suctioning, instrumentation and tumour oozing/desloughing impaired continuous tracking and required further user interaction. The distance and angulation of the camera as well as the depth of ICG have been found to influence the signal and need consideration in further protocols^40^. Furthermore, while other surgical camera manufacturers offer similar systems for ICG visualization by NIR, camera design and performance investigation has shown previously significant inter-manufacturer variation between signal and ICG concentration (including the tendency of some systems to boost the images for human interpretation) which limits the generalizability of these findings until further (ongoing) validation is performed^41^. While selection of the specific timepoints for analysis (e.g. 100 s post-peak timepoint) is somewhat arbitrary, they do provide milestones for statistical analysis enabling comparisons between lesions and adjacent healthy tissue as control. Statistical curve analysis is currently not calculated in real time and is limited to one curve per ROI. Simultaneous synthesis and dissection of multiple curves may further discern tumour heterogeneity. Although the automatic classifier diminished the differentiation ability of our metrics when compared to surgeon-based classification, better classification algorithmic methods may to some extent replace operator input. Other tracking algorithms exist and further software development may address field of view interruptions.

This endeavour has nonetheless shown the feasibility of ICG with NIR-illumination for continuous quantitative FI assessment. Software assessment of these perfusion curves has yielded metadata which could potentially be used to identify cancer in human patients. The previous focus on spot readings may alleviate the need for concurrent injection and recording but has overlooked the potential for incorporating dynamic ICG flow patterns to improve confidence of interpretation. Equally incorporation of computer-assistance can help and even potentially replace the need for human observer visualization promising near real-time assessment of lesions during procedures with minimal user interaction as a ‘digital biopsy’ enabled through advances in computer processing, artificial intelligence and broad cloud-based datasets^42^.
